# Are there any benefits of exercise training in cancer cachexia?

**DOI:** 10.1007/s13539-012-0067-5

**Published:** 2012-05-08

**Authors:** Josep M. Argilés, Sílvia Busquets, Francisco J. López-Soriano, Paola Costelli, Fabio Penna

**Affiliations:** 1 Departament de Bioquímica i Biologia Molecular, Facultat de Biologia, Universitat de Barcelona, Diagonal 643, 08028 Barcelona, Spain; 2 Institut de Biomedicina de la Universitat de Barcelona, Barcelona, Spain; 3 Dipartimento di Medicina e Oncologia Sperimentale, Università di Torino, Torino, Italy

## Abstract

Cancer cachexia is a complex syndrome characterized by inflammation, body weight loss, muscle, and adipose tissue wasting that is responsible for the death of a considerable percentage of cancer patients. In addition, during cachexia muscle strength and endurance are dramatically reduced, limiting the ability to perform daily activities and severely affecting the patient’s quality of life. Different studies have emphasized that a single therapy may not be completely successful in the treatment of cachexia. Beyond pharmacological strategies, exercise training has been suggested as a promising countermeasure to prevent cachexia, in order to restore both strength and endurance, depending on the type of exercise. Unfortunately, a small number of studies, in both clinical and experimental settings, have been performed to date. Moreover, when considering exercise in cancer, several factors have to be taken into consideration, in particular those alterations that could limit the capacity to perform exercise and consequently the resulting beneficial or detrimental effects. This editorial is aimed at stimulating the debate on the suitability of including exercise training in a multi-functional approach against cachexia taking into consideration both limitations and advantages.

Cachexia is a multiorganic syndrome associated with cancer, characterized by inflammation, body weight loss (at least 5 %) and muscle and adipose tissue wasting [1]. The abnormalities associated with cachexia include alterations in carbohydrate, lipid and protein metabolism, often associated with anorexia [1–3]. Cachexia occurs in the majority of subjects with cancer before death, and it is responsible for the deaths of approximately 20 % of cancer patients [4], although the percentage may be even higher, depending on cancer type [5]. In addition, survival of patients affected by different types of neoplasias is clearly dependent on the presence of weight loss [6]. Therefore, cachexia prevention represents an important factor in the treatment of cancer patient, affecting not only survival but also the tolerance to anti-cancer treatment, quality of life and, ultimately, sanitary costs. It is thus clear that there is both a medical and a social need for the treatment of cancer cachexia. Although anorexia represents a very important factor in the development of cachexia, it has to be pointed out that in many cases the use of total parenteral nutrition does not stop the loss of body weight [7]. It seems, therefore, quite evident that metabolic disturbances present in the host have a definitive role in the development of cachexia [2, 7]. Among these, increased energy inefficiency, insulin resistance, abnormal carbohydrate metabolism, adipose tissue dissolution, hyperlipidemia, and muscle wasting are the most relevant. Because metabolic alterations often appear soon after the onset of tumor growth, the scope of appropriate treatment, although not aimed at achieving immediate eradication of the tumor mass, could influence the course of the patient’s clinical state or, at least, prevent the steady erosion of dignity that the patient may feel in association with the syndrome. This would no doubt contribute to improving the patient’s quality of life and, possibly, prolong survival.

Different studies have emphasized that a single therapy may not be completely successful in the treatment of cachexia. From this point of view, treatments involving different combinations are more likely to be successful [8]. A very interesting phase II study, involving the administration of antioxidants, pharmaconutritional support, progestagen, and anti-cyclooxygenase-2 drugs, showed efficacy and safety in the treatment of patients with advanced cancer of different sites suffering cachexia [9]. Based on the results of the Phase II study a randomized Phase III study started in 2005 with the aim to include more than 300 cachectic cancer patients and it is still in progress. These data clearly reinforce the use of these multitargeted therapies in the treatment of the cachexia–anorexia syndrome in cancer. Beyond pharmacological strategies, exercise training has been suggested as a promising countermeasure to prevent cachexia [10]. Unfortunately, a small number of studies, in both clinical and experimental settings, have been performed in order to define the effectiveness of exercise against cachexia. The rationale for the use of exercise is quite simple; it has been demonstrated that during cachexia muscle strength and endurance are dramatically reduced [11–14]. Such alterations severely limit the ability to perform daily activities and consequently compromise the patient’s quality of life. Exercise training, on the contrary, is able to increase both strength and endurance in healthy conditions, depending on the type of exercise. Moreover, it has been proven to act as an excellent anabolic drive for skeletal muscle in combination with anabolic steroid or other muscle anabolic drugs [15].

## Limitations to exercise in cancer

When considering exercise in cancer, several factors have to be taken into consideration, in particular those alterations that could limit the capacity to perform exercise and consequently the resulting beneficial or detrimental effects. Actually, many cancer patients suffer from chronic fatigue, either from the disease itself or its treatment, the latter being a confounding factor that limits exercise practice. As a matter of fact, recent data from our laboratory suggest that exercise may be not advisable when anemia is present. Indeed, as can be seen in Table 1, when tumor-bearing mice suffering from a significant decrease in hematocrit are exercised (mild endurance training), their condition worsens. It was reported that anemia is a frequent feature of patients with cancer cachexia, contributing to weight loss, reduced exercise capacity, and altered energy homeostasis [16]. The incidence of anemia varies with tumor type, stage and patient’s age: up to one-third of cancer patients are anemic at diagnosis [17] and chemotherapy can even increase this number. Cancer-associated anemia can thus be considered a negative prognostic factor for survival, regardless of tumor type [18]. The mechanisms by which anemia may contribute to the onset and progression of cachexia still need to be elucidated, however, in the design of a therapeutical approach against cachexia that include exercise training, it is mandatory to correct anemia first, in order to rescue the tolerance to the exercise.

**Table 1:** Body and muscle weight in control (*n* = 6), colon26-bearing (C26, *n* = 8), exercised (*n* = 6, 14 m/min 45 min, 5 days/week on a treadmill) and C26 exercised (*n* = 8)

	Controls	C26	Exercise	C26 exercise
Initial b.w. (g)	21.06 ± 1.02	20.88 ± 0.79	20.82 ± 1.15	20.76 ± 0.60
Final b.w. (g)	23.94 ± 1.29	20.06 ± 1.37	23.84 ± 1.34	17.42 ± 1.73
Δ b.w. (g)	+2.88 ± 0.57	−0.82 ± 1.23**	+3.02 ± 0.63	−3.34 ± 1.60***^,^ ****
GSN mass (mg/100 g i.b.w.)	572 ± 25	471 ± 49**	581 ± 26	408 ± 54***^,^ ****
Hematocrit (%)	51 ± 4	42 ± 7 *	54 ± 2	44 ± 6 *

The hematocrit was measured collecting the blood by means of cardiac puncture from anesthesized mice before sacrifice. Data are expressed as mean±standard deviation

*p < 0.05 vs. control; **p < 0.01 vs. control; ***p < 0.001 vs. control; ****p < 0.05 vs. C26

**Fig. 1:**
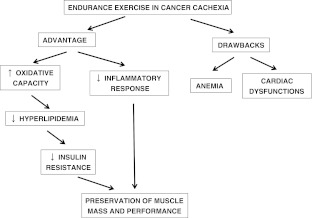
Diagram depicting the effects of endurance exercise in cancer cachexia

Although relevant, anemia is not the unique factor limiting exercise capacity in cancer, since recent data have reported functional and structural cardiac alterations in tumor-bearing mice [19]. Anemia and cardiac dysfunctions likely act synergistically lowering the threshold of exercise intensity that discriminate between the beneficial adaptations induced by mild exercise and the deleterious effects of strenuous exercise (Fig. 1). Thus, the exercise dose is a crucial point to be carefully evaluated on a single patient basis. Nonetheless, the mode of exercise to be performed in a cancer patient population is very important, although a consensus is still lacking. Considering the maintenance of muscle mass as the primary goal to be achieved in cachexia, resistance rather than endurance exercise appears to be preferred. Indeed, while the former exerts an anabolic action, the latter stimulates oxidative metabolic adaptations with little changes in muscle mass. However, the resistance induced anabolism does not necessarily imply an anticatabolic effect. Indeed, resistance exercise in healthy conditions stimulates the Akt/mTOR signaling pathway [20], this being reported to be unaffected or even hyperactivated in tumor-bearing animals [21], suggesting the uselessness of Akt stimulation to prevent muscle wasting in cancer cachexia. On the contrary, mild endurance exercise might counteract the reduction of oxidative capacity found in experimental cachexia [22]. The stimulation of oxidative metabolism could directly prevent the hyperlipidemia, and consequently ameliorate the insulin resistance. Finally, endurance exercise induces physiological adaptations resulting in the attenuation of the inflammatory response [23] (Fig. 1). As a result, endurance exercise has been the exercise mode of choice in the majority of atrophy countermeasure studies performed to date. A recent meta-analysis [24] reported differential effects exerted by exercise, depending on the pathology considered and the type of exercise performed: a global positive effect of exercise was observed when all the pathologies considered were pooled together. It is interesting to speculate that lower than previously recognized volumes of exercise are quite likely to have a measurable and positive impact in neutralizing muscle loss if practiced diligently and started at early stages of the disease, even in clinical cancer populations. Finally, the relevance of the nutritional state of the patients should be taken into account. In this regard, the potential benefits of exercise can be completely abrogated if the organism does not have a good nutrient availability that would allow the effective counteraction of the wasting pattern. Thus, the management of cancer cachexia will probably be improved by a multi-functional approach. In this regard, the effects of specific anabolic/anticatabolic drugs associated with adequate nutritional support could be potentiated by increased muscle use with moderate-to-high endurance.
